# How Should We Identify Home‐ and Community‐Based Services (HCBS) in Medicaid Claims Data? An Evaluation and Synthesis of Algorithms

**DOI:** 10.1111/1475-6773.70164

**Published:** 2026-09-16

**Authors:** Vimbainashe Dihwa, Eric T. Roberts, Eliza Macneal, Kenton J. Johnston, Ari Ne′eman

**Affiliations:** ^1^ Perelman School of Medicine and Leonard Davis Institute of Health Economics University of Pennsylvania Philadelphia Pennsylvania USA; ^2^ Washington University School of Medicine St. Louis Missouri USA; ^3^ Harvard T.H. Chan School of Public Health Boston Massachusetts USA

**Keywords:** administrative claims data, HCBS, T‐MSIS analytic files

## Abstract

**Objective:**

To assess existing algorithms for measuring Home‐ and Community‐Based Services (HCBS) in Medicaid claims, and to develop and evaluate a unified, modified algorithm.

**Study Setting and Design:**

Using Transformed Medicaid Statistical Information System Analytic Files (TAF), we compared two existing algorithms for identifying and classifying 1915(c) waiver and state plan HCBS. Informed by that analysis, we developed a third, unified and modified algorithm. Using each algorithm, we constructed annual state‐level estimates of HCBS use per 100,000 residents, which we compared from 2021 to 2023.

**Data Sources and Analytic Sample:**

National 20% sample of TAF data. The sample included continuously enrolled, full‐benefit Medicaid beneficiaries age 19 or older in 2021 for primary analyses, and 2022–2023 to compare counts over time.

**Principal Findings:**

The existing algorithms showed a high level of concordance (observed agreement, *P*_*o*_ = 93% and kappa = 0.77). Disagreement resulted from missing or inconclusive program type codes, differences in service‐related codes used to classify HCBS, and differences in classifying claims for capitated payments. Our algorithm addressed some of this disagreement with the novel use of waiver identification numbers and modification of some TAF data elements and codes. HCBS user counts were mostly consistent across algorithms, with our algorithm identifying the broadest population of users. However, some states exhibited inconsistent counts depending on the algorithm, implausibly high counts (Pennsylvania and Illinois), or implausibly low counts (Mississippi). In several states, HCBS user counts varied substantially from 2021 to 2023 (> 50% in absolute value), suggesting changes in data quality or reporting in those states across years.

**Conclusions:**

Our algorithm resolves some discordances between two existing algorithms for measuring HCBS use in TAF. However, in certain states and years, inconsistent or implausible HCBS user counts suggest variations in data reliability and coding patterns that could limit accurate measurement of HCBS, regardless of the algorithm used.

## Key Points

- What is known on the topic.
  ○ Medicaid Home and Community‐Based Services (HCBS) are an important and growing mechanism for delivering Long‐Term Services and Supports (LTSS).
  ○ HCBS can be covered through state plans or waiver authorities.
  ○ Researchers have been developing approaches for identifying and classifying HCBS in claims data to support research that informs LTSS‐relevant health policy and practice.
- What this study adds.
  ○ We assessed two existing algorithms for identifying and classifying state plan and 1915(c) waiver HCBS in Medicaid claims data.
  ○ We developed a third algorithm that unifies the two existing approaches and introduces novel modifications (including the use of waiver identification numbers) to clarify classification for discordant claims.
  ○ Each algorithm has strengths and trade‐offs that researchers should keep in mind when measuring HCBS in TAF, especially given state and year variations in TAF data quality and reporting.

## 1 Introduction

Medicaid Home and Community‐based Services (HCBS) are an important type of Long‐Term Services and Supports (LTSS), accounting for the highest proportion of users (86.2%) and spending (64.6%) [1] among Medicaid enrollees using LTSS in 2021. As an alternative to institutional long‐term care, HCBS programs allow Medicaid beneficiaries with significant physical or mental disabilities to receive services within their homes or in a community‐based residential setting [2]. HCBS encompass a broad range of supports for daily living, such as personal care, supported employment, day services, respite care, and other ongoing supports.

Although HCBS are widely utilized among Medicaid beneficiaries who use LTSS, there are state‐specific variations in operational definitions and eligibility criteria [3] for HCBS. States have substantial flexibility in what types of HCBS they pay for and the financing authorities they use to deliver HCBS. Specifically, Medicaid HCBS is covered through a combination of state plan and waiver authorities, with different states opting for different financing structures for the same services. Services covered under a Medicaid state plan must be provided to all individuals who meet functional eligibility criteria for them, whereas waivers allow states to cap enrollment and establish waiting lists for services [4].

In 2021, an estimated 2.6 million people received Medicaid HCBS services in the US [5]. 48.1% of Medicaid HCBS expenditures were covered by 1915(c) HCBS waiver programs that year [3], with nonwaiver services covered by a range of state plan authorities. State plan financing authorities include the core Section 1905(a) Medicaid state plan benefit and optional state plan authorities (e.g., 1915(i), (j), or (k)). In addition, Medicaid section 1915(b) and 1115 waivers allow states to deliver HCBS through managed care programs. How these various authorities are used to finance and deliver HCBS differs across states, leading to considerable challenges in identifying HCBS in a consistent manner within national Medicaid administrative data.

Examining the use of HCBS and the extent to which these services address Medicaid beneficiaries' LTSS needs remains an ongoing area of research [6, 7, 8, 9]. Accurate and comparable measurement of HCBS use and classification by 1915(c) and state plan coverage are imperative for advancing this research and informing Medicaid long‐term care policy. With those goals in mind, researchers have been developing approaches to identify and classify HCBS in Transformed Medicaid Statistical Information System Analytic Files (TAF) [4, 10, 11, 12], national Medicaid administrative data that include detailed information on service utilization. TAF data were intended to overcome limitations of predecessor Medicaid data, known as Medicaid Analytic eXtract (MAX) files [12]. However, research has found that TAF data still present analytical challenges due to missingness and variability in data quality by state and year, with variables such as HCBS taxonomy codes considered unreliable [13]. Additionally, there are trade‐offs in the benefits and limitations of using specific TAF data elements to measure HCBS utilization.

This paper makes three major contributions. First, we compare two existing algorithms for identifying and classifying HCBS as either 1915(c) waiver or state plan services. We examine cases where these algorithms differently identify or classify HCBS to understand the strengths and limitations of each approach. Second, we develop a third, unified algorithm that preserves the strengths of the existing algorithms while incorporating modifications informed by our comparison of existing algorithms. Third, we evaluate the performance of all three approaches by using each algorithm to produce annual state‐level estimates of total HCBS users, and users of 1915(c) waiver services and state plan HCBS, which we compare from 2021 to 2023. This paper also adds to efforts to support the reproducibility of TAF‐based research [14] by highlighting considerations for researchers working with TAF data to measure HCBS use.

## 2 Methods

### 2.1 Data and Sample

TAF other service (OT) claims files were used in this study. OT files contain service‐level data on care received by Medicaid beneficiaries, including HCBS. These data include codes for the Medicaid program or financing authority under which a service was provided (e.g., program type code), waiver information, broad service classification codes (e.g., type of service codes), and detailed service‐level codes (e.g., procedure codes). These data include encounter records submitted by Medicaid managed care plans and fee‐for‐service claims from states that directly administer Medicaid benefits. For brevity, we refer to both types of records as *claims*.

Claims from all 50 states and the District of Columbia were included for a 20% random sample of continuously enrolled, full‐benefit Medicaid beneficiaries who were aged 19 or older in the analysis year. The sample includes (but is not limited to) dually‐eligible Medicare‐Medicaid beneficiaries. Residents of US territories (e.g., Puerto Rico) were excluded. Our primary analyses use 2021 data; we included 2022–2023 data to compare changes in counts of HCBS users over time. Final OT files were used for 2021–2022, while preliminary OT data were used for 2023. Counts were measured per 100,000 residents, and the data source for our state‐level population estimates was 2020 Census data [15].

### 2.2 Study Measures: HCBS

We measured Medicaid‐funded HCBS, broadly defined as Medicaid‐covered services that serve as alternatives to institutional care and allow individuals to live independently at home or in community‐based settings [2]. Each algorithm measures two types of HCBS services in the claims data: home health services to meet medical needs and human services to support daily living [16]. Health services include skilled nursing care, durable medical equipment, case management, home health care, and other services that are necessary for treating medical conditions. Human services include home‐delivered meal programs, transportation, personal care, and other services that allow individuals with functional limitations to live independently. Analytic code, accompanying files, and instructions for running the code are available online on a Figshare repository at https://doi.org/10.6084/m9.figshare.32984042 [17].

### 2.3 Analyses

There were three stages of analysis in this study. First, we conducted comparative analyses to assess how two existing algorithms identify HCBS in TAF data and classify these services as either 1915(c) waiver HCBS (also referred to herein as waiver HCBS) or state plan HCBS. We then developed a third, unified and modified algorithm based on our findings from the first stage of analysis. Finally, we compared all three algorithms by applying them to the TAF to calculate counts of HCBS users per 100,000 residents by state, in aggregate and stratified by waiver and state plan HCBS. The TAF data elements in each algorithm are presented in Table S1.

#### 2.3.1 Comparing Existing Approaches

We first compared two existing algorithms for identifying and classifying HCBS, evaluating areas of concordance and discordance between them and noting the trade‐offs, strengths, and limitations of each in identifying HCBS claims and classifying them as waiver or state plan HCBS.

Algorithm 1 (Mathematica) was implemented by applying Mathematica's methodology documented in the Centers for Medicare and Medicaid Services (CMS)'s 2024 guidance for identifying and classifying Medicaid long‐term services and supports (LTSS) [11]. This algorithm uses a hierarchical approach that prioritizes Medicaid financing codes. A claim is classified as waiver HCBS based on the program type code, waiver type code, and HCBS service code (in that order), with services lacking sufficient information at one stage evaluated at the next. Claims not identified as waiver HCBS are then assessed against the criteria for classifying state plan HCBS, again prioritizing financing codes over service‐related codes. Algorithm 1 is described in more detail in Table S2.

Algorithm 2 (Wang et al.) was applied by using the methodology published by Wang, Qi, and Konetzka (2024) [12]. This algorithm classifies a claim as waiver HCBS when two conditions are met. First, a claim is identified as HCBS through a combination of three approaches using type of service (TOS) codes, HCBS taxonomy codes, and procedure codes mapped to HCBS taxonomy codes from legacy MAX data. Second, the claim should also have a program type code or a waiver type code indicating a 1915(c) waiver service. State plan HCBS claims are identified using only TOS codes, restricted to claims that do not have a program type code or waiver type code indicating a waiver service. Algorithm 2 is described in Table S3.

#### 2.3.2 Developing Algorithm 3

**FIGURE 1:**
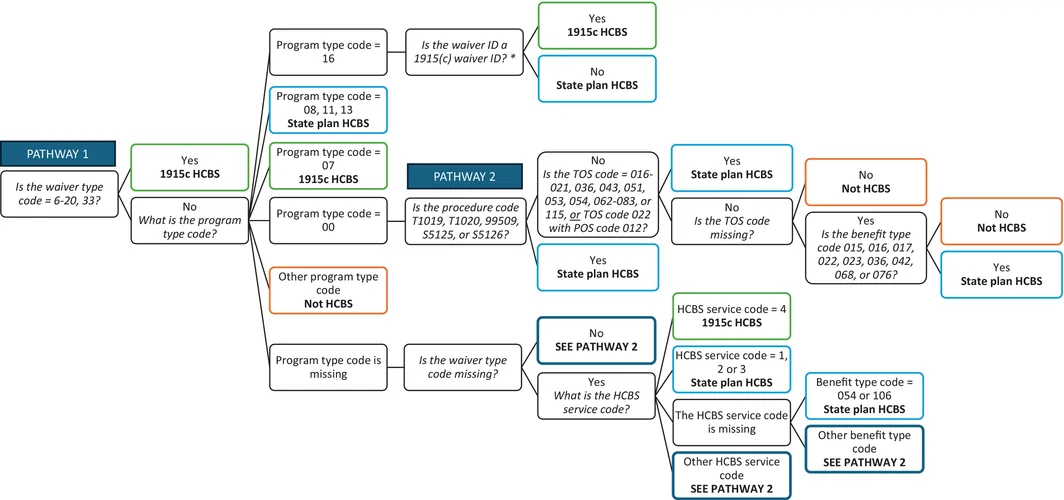
Logic of Algorithm 3. *Waiver IDs: Claims with program type code 16 were classified as waiver or state plan HCBS based on the Waiver ID. We determined that 1915(c) waiver IDs followed the format “XX.###,” where XX is a state code and ### is a 3‐ to 8‐digit number, and we confirmed that those waiver IDs were provided through 1915(c) waiver authorities by using the list of state waivers from the Medicaid.gov website [18].

Informed by our comparison of the two existing algorithms, we constructed Algorithm 3, maximizing the strengths of existing approaches, with modifications to address discordance in the identification and classification of HCBS. Figure 1 details Algorithm 3's logic.

Consistent with the existing algorithms, Algorithm 3 classifies claims as waiver HCBS when they have either waiver type codes indicating 1915(c) services (codes 06‐20, 33) or a program type code indicating waiver HCBS (code 07). However, we also made modifications. Unlike Algorithm 1 (Mathematica), our algorithm accepts a waiver type code or program type code as sufficient on its own for classification instead of prioritizing program type code over waiver type code. And unlike Algorithm 2 (Wang et al.), our algorithm does not require additional information from a TOS code, procedure code, or HCBS taxonomy code (in addition to the waiver type codes or program type code) to classify those services as waiver HCBS.

These modifications allowed us to identify 1915(c) waiver services with a relevant waiver type code even when accompanied by a program type code that does not indicate 1915(c) services (e.g., program type code 16, which can be used for 1915(c) waiver and 1915(j) state plan services). To verify the classification of claims with program type code 16, we introduced the novel use of Waiver IDs, which are assigned by CMS for Medicaid waiver programs [19]. We also examined the Waiver IDs on claims with a program type code other than 07 accompanied by a 1915(c)‐indicating waiver type code to inform our decision to classify these as waiver HCBS.

Most HCBS waivers have a distinct format differentiating them from other types of waivers. Accordingly, we first identified Waiver IDs with the common format “XX.###,” where XX is a 2‐character state code and ### is a 3‐ to 8‐digit number, and then used the Medicaid.gov website to confirm that the waiver IDs using this format were for 1915(c) waivers. The Waiver IDs used in this study are listed in Table S6. Although Waiver IDs helped with confirming the classification of waiver HCBS claims, researchers should note that Waiver IDs may not follow the same format over time due to data quality issues or name changes when waivers are amended or renewed (such as an added suffix code). Researchers should carefully review this variable to ensure that only Waiver IDs relevant to 1915(c) services are included and that amendments to these waivers are not inadvertently excluded.

Consistent with Algorithm 1, our algorithm uses other data elements to classify waiver and state plan HCBS when the program type and waiver type codes are ambiguous or missing. For example, we use HCBS service code 4 to identify waiver HCBS when the program type code and waiver type code are not reported and use benefit type codes to identify state plan HCBS when program type, waiver type, and HCBS service codes are missing. Procedure codes, TOS codes, and benefit type codes are used to identify state plan HCBS when the program and waiver type code are missing or ambiguous. To classify state plan HCBS, we modify our unified algorithm by using the criteria described in Algorithm 1 while expanding the TOS codes used to include the additional codes used to identify state plan HCBS in Algorithm 2.

We do not include hospice services given the differences in the policy and regulatory oversight for hospice services when compared to HCBS. We also exclude HCBS taxonomy codes due to data quality issues [4, 12]. The specific TAF data elements used for Algorithm 3 are in Table S5, and descriptions of those TAF data elements are in Table S6.

#### 2.3.3 Comparing the Three Algorithms for Identifying and Classifying HCBS

Using TAF OT files from 2021, we compared the three algorithms by constructing state‐level counts of HCBS users as rates per 100,000 residents, in aggregate and for users of waiver and state plan services. To assess the performance of these algorithms across different years, we also used each algorithm to construct time trends of HCBS user counts in 2022 and 2023 relative to 2021. We used DQ Atlas assessments [20] to note which states had unreliable waiver HCBS user counts in a year.

#### 2.3.4 Supplementary Analyses

To our knowledge, there is no gold standard external (i.e., non‐TAF) benchmark for state‐level HCBS use. However, we conducted two supplementary analyses to check for inconsistencies in TAF data that may reflect underlying data quality issues. First, we compared counts of full Medicaid enrollees in the TAF Demographic and Eligibility File (D&E file) to state‐level counts of full‐year equivalent Medicaid enrollment reported by the Medicaid and CHIP Payment and Access Commission (MACPAC) [21]. The D&E file reports a census of Medicaid enrollees and was the basis for how we identified our sample. For each state, we also indicated the DQ Atlas data quality assessment for the eligibility group code [22], identifying states with potentially unreliable HCBS data. Inconsistencies in Medicaid enrollee counts may suggest TAF data limitations in identifying the Medicaid‐enrolled population using HCBS.

Second, noting substantial variations in TAF data elements used to classify HCBS using TAF OT files, we used the D&E file to construct 1915(c) waiver participant counts. We constructed two different counts using a variable that indicates that a beneficiary was enrolled in a 1915(c) waiver for at least a month and another variable that flagged 1915(c) waiver coverage. The first count was compared to HCBS data from KFF [23] and the second count was compared to HCBS data from the DQ Atlas [20].

## 3 Results

### 3.1 Comparing Existing Approaches

We found that Algorithms 1 and 2 agreed on the identification and classification of 93% of the cases (observed agreement, *P*_*o*_ = 0.93), indicating a high level of concordance at the claim level. After accounting for the agreement expected by chance, Cohen's kappa statistic was 0.77, representing a substantial level of concordance according to Landis and Koch's classification [24, 25]. The concordance calculations are presented in Table S4. Areas of agreement are shown as Cases 1, 5, and 9 in Table 1. We found that 97% of claims that were identified as non‐HCBS by Algorithm 1 were also considered non‐HCBS by Algorithm 2 (Case 1). Case 5 shows that 71% of claims classified as state plan HCBS by Algorithm 1 were also classified as state plan HCBS using Algorithm 2. Similarly, 86% of claims that Algorithm 1 classified as waiver HCBS were also classified as waiver HCBS using Algorithm 2 in Case 9.

**TABLE 1:** Comparative performance analysis of Algorithm 1 (Mathematica method) and Algorithm 2 (Wang et al. method) in identifying and classifying waiver and state plan HCBS, 2021.

Total %^a^ Row % ^b^ Column % ^c^	Algorithm 1 (Mathematica method)		
	Not HCBS	State plan HCBS	Waiver HCBS
**Algorithm 2 (Wang et al. method)**			
**Not HCBS**	Case 1	Case 2	Case 3
	80%	1%	1%
	97%	2%	1%
	97%	14%	12%
**State Plan HCBS**	Case 4	Case 5	Case 6
	2%	7%	0%
	19%	80%	1%
	2%	71%	1%
**Waiver HCBS**	Case 7	Case 8	Case 9
	1%	2%	6%
	9%	18%	72%
	1%	15%	86%

*Note:* Cell, column, and row percentages may not sum to 100% due to rounding. Each cell is labeled as a “Case” to simplify discussion of the results. For example, the first cell is Case 1.

Abbreviation: HCBS, home‐ and community‐based services.

^a^ Total % indicates what percentage of the entire sample (grand total of all the cells) is in a specific cell.

^b^ Row % (shown in blue) refers to the cell count divided by the total count of a specific row, indicating the percentage claims in the row that fall under a specific column category.

^c^ Column % (shown in green) refers to the cell count divided by the total count in a specific column, indicating the percentage of claims in the column that belong in a particular row category.

### 3.2 Differences Between Algorithms 1 and 2

Discordance between Algorithms 1 and 2 occurred for two reasons: (1) disagreement in whether a claim was identified as HCBS (Cases 2, 3, 4, and 7 in Table 1); and (2) disagreement in classification of a claim as waiver or state plan HCBS (Cases 6 and 8). A summary of these cases, the states they occur in most often, and the approaches employed in our modified unified algorithm (Algorithm 3) to address discordance are in Table 2.

**TABLE 2:** Cases of disagreement between Algorithm 1 (Mathematica method) and Algorithm 2 (Wang et al. method).

	Cases^a^	Algorithm 1 (Mathematica)	Algorithm 2 (Wang et al.)	Our findings			
				Pattern of discordance^b^	Common states^c^	Algorithm 3 solution to discordance^d^	Majority classification in Algorithm 3 (%)^e^
Disagreement in identifying HCBS	Case 2	State plan	Not HCBS	Personal care service procedure codes	TX (80%), FL (10%)	Use personal care service procedure codes to identify state plan HCBS.	State plan (99%)
	Case 3	Waiver	Not HCBS	Capitated payments	IL (73%), AL (13%)	Use finance codes on their own to identify waiver HCBS.	Waiver (100%)
	Case 4	Not HCBS	State plan	1905(a) services	CA (33%), MA (18%)	Use expanded set of type of service codes from Wang et al.	State plan (83%)
	Case 7	Not HCBS	Waiver	Program type code 00 (no special program) and 1915(c) waiver type code (6‐20, 33)	PA (72%), NC (12%)	Use waiver type code to classify waiver HCBS regardless of program type code.	Waiver (100%)
Disagreement in classifying waiver HCBS	Case 6	Waiver	State plan	Missing program type code, missing waiver type code, service provided under 1915(c) waiver (HCBS service code 4)	TX (100%)	Use HCBS service code to classify waiver HCBS in absence of program type code and waiver type code.	Waiver (100%)
	Case 8	State plan	Waiver	Program type code 16 (1915(j) services) and 1915(c) waiver type code (6–20, 33)	PA (39%), WI (17%)	Use waiver type code and Waiver ID to classify 1915(j) under waiver authority as waiver HCBS.	Waiver (100%)

Abbreviations: HCBS, home‐ and community‐based services. Waiver IDs, Waiver identification numbers assigned by Centers for Medicare and Medicaid Services (CMS) to specify approved Medicaid waiver or demonstration programs.

^a^ *Cases*. As labeled in Table 1. The specific cases of disagreement in the identification and classification of HCBS between Algorithms 1 and 2.

^b^ *Pattern of discordance*. Our findings on the types of claims that were most common among claims for which a particular type and case of disagreement existed. This was determined using novel approaches that were later incorporated into Algorithm 3.

^c^ *Common states*. The states in which a particular type and case of algorithm disagreement occurred the most, and the percentage of claims that were associated with that state.

^d^ *Algorithm 3 solutions to discordance*. The method that we used to classify a claim as state plan HCBS, waiver HCBS, or not HCBS in each case. This includes our novel solutions using Waiver IDs and modifying the specific codes of some of the data elements used by existing algorithms. In some cases, we used the same method used by one of the existing algorithms after assessing its performance (Case 2, Case 3, Case 4, and Case 6).

^e^ *Majority classification in Algorithm 3 (%)*. This column indicates the percentage of claims under this case that Algorithm 3 classifies as either 1915(c) waiver or state plan HCBS. For example, in case 2, Algorithm 3 classified 99% of claims as state plan HCBS.

#### 3.2.1 Disagreement in Identification of HCBS

Case 2 shows that 14% of the claims that were classified as state plan HCBS by Algorithm 1 were identified as non‐HCBS by Algorithm 2. These claims were identified as state plan HCBS by Algorithm 1 but not Algorithm 2 because 85% did not have TOS codes indicating state plan HCBS but had procedure codes indicating state plan personal care services, and 14% had a program type code indicating 1915(i), 1915(k), or state plan 1915(j) services. Therefore, Algorithm 3 addressed this case of discordance by classifying these claims as state plan HCBS.

For Case 3, we see that 12% of claims that Algorithm 1 classified as waiver HCBS were identified as non‐HCBS by Algorithm 2. 77% of these claims had TOS codes for capitated payments, with Illinois, Alabama, Colorado, Wisconsin, and Nebraska accounting for most of these cases. Although these claims also had a waiver type code or program type code indicating waiver services, Algorithm 2 did not classify them as waiver HCBS because an HCBS‐specific TOS code or procedure code was not present. To address this discordance, Algorithm 3 classifies these claims as waiver HCBS based solely on the presence of a relevant program type code or waiver type code.

In Case 4, 2% of the claims that Algorithm 1 identified as non‐HCBS were classified as state plan HCBS by Algorithm 2. Of these claims, 91% had a TOS code used by Algorithm 2, but not by Algorithm 1, to identify state plan HCBS, while 9% had a TOS code used by both algorithms but a program type code other than 08, 11, 13, or 16. To address this discordance, Algorithm 3 used the broader set of TOS codes from Algorithm 2 while retaining the structure of Algorithm 1, wherein TOS codes are only used for classification of state plan HCBS if the program type code is missing or indicates no special program (code 00). This modification allowed us to classify 83% of claims in Case 4 as state plan HCBS.

Case 7 shows that 1% of claims identified as non‐HCBS by Algorithm 1 were classified as waiver HCBS by Algorithm 2. Of these, 98% had a program type code indicating no special program (code 00) accompanied by a 1915(c) waiver type code. Pennsylvania and North Carolina accounted for most of these cases. Informed by our finding that 97% of these claims also had a 1915(c)‐related Waiver ID, this case of discordance was resolved in Algorithm 3 by classifying these claims as waiver HCBS.

#### 3.2.2 Disagreement in Classification of HCBS

Case 6 shows that 1% of claims classified as waiver HCBS by Algorithm 1 are classified as state plan HCBS by Algorithm 2. This case consisted entirely of a small set of claims in Texas with no program type code, no waiver type code, and HCBS service code 4 indicating a service provided under a 1915(c) waiver. Algorithm 3 classifies these claims as waiver HCBS.

In Case 8, 15% of claims that are classified as state plan HCBS by Algorithm 1 are considered waiver HCBS by Algorithm 2. We found that 78% of these claims had program type code 16, which classified them as state plan HCBS under Algorithm 1. However, 100% of these claims also had a waiver type code indicating a 1915(c) service, and 99% had a 1915(c)‐related Waiver ID. Accordingly, Algorithm 3 classifies these claims as waiver HCBS.

### 3.3 Comparing the Three Algorithms for Identifying and Classifying HCBS

**FIGURE 2:**
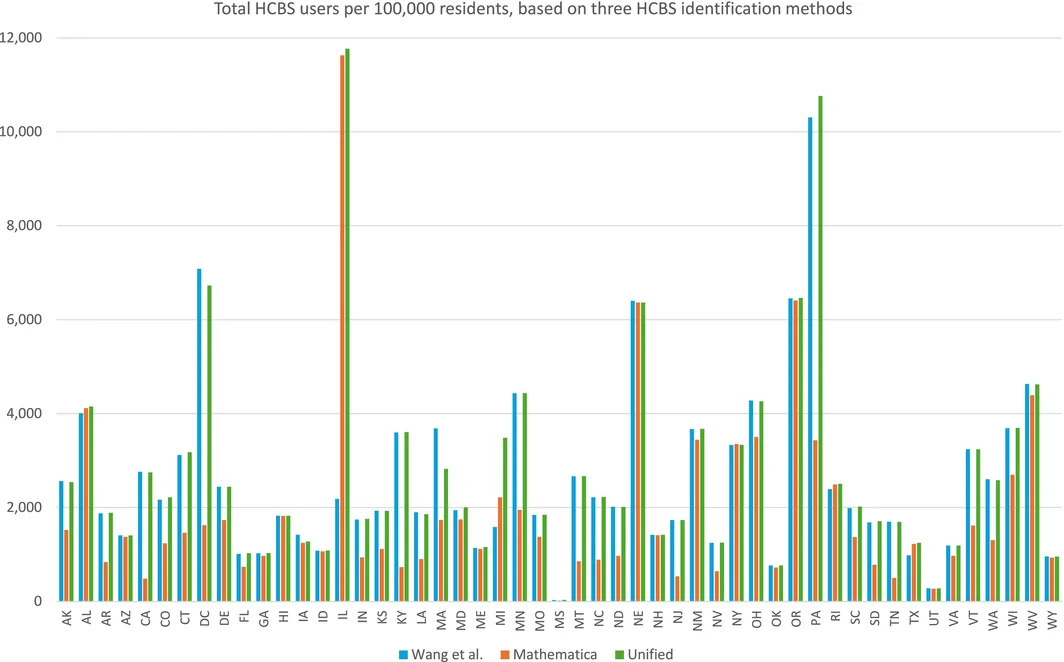
Overall state HCBS user counts per 100,000 residents. The DQ Atlas did not report assessments of the reliability of data for 1915(c) waiver HCBS participation by state in 2021 [20].

Figure 2 shows state‐level HCBS counts per 100,000 residents for each algorithm. Waiver HCBS users and state plan HCBS users are shown in Figures S1 and S2, respectively. Overall results were mostly consistent across algorithms, with Algorithm 3 identifying the broadest population of HCBS users. However, the algorithms yielded implausibly large or inconsistent counts of HCBS users in Pennsylvania and in Illinois, and implausibly low counts in Mississippi, suggesting underlying data quality concerns in those states' 2021 TAF data. Additionally, inconsistent counts in several other states (e.g., California, Kentucky, Minnesota), depending on the algorithm, suggest trade‐offs among algorithms based on their prioritization of data elements and variations in the reliability and coding patterns of those elements by state.

Generally, Algorithm 2 identified a broader set of state plan HCBS than Algorithm 1 (Figure S2). This reflected a wider set of TOS codes used by Algorithm 2 to identify state plan HCBS. As the unified approach, Algorithm 3 generally exhibited the broadest definition of HCBS. However, due to some of our modifications, Algorithm 2 identified more state plan HCBS users than both Algorithms 1 and 3 in a few states (DC, MA, and MI), while Algorithm 1 identified more in others (AL, OK, and PA). In the former case, this was because of Case 4 claims. Algorithm 3 implements the broader set of TOS codes used by Algorithm 2 to identify state plan HCBS but maintains the Algorithm 1 approach of using TOS codes to identify state plan HCBS only among non‐program‐based claims. As a result, some Case 4 claims were not identified as state plan HCBS by Algorithm 3. In the latter case, this was because the state had many claims falling under Case 8, meaning they were classified as state plan HCBS by Algorithm 1 but classified as 1915(c) waiver HCBS by Algorithms 2 and 3 due to having a waiver type code of 6‐20 or 33, yielding higher counts of state plan HCBS users when using Algorithm 1.

**FIGURE 3:**
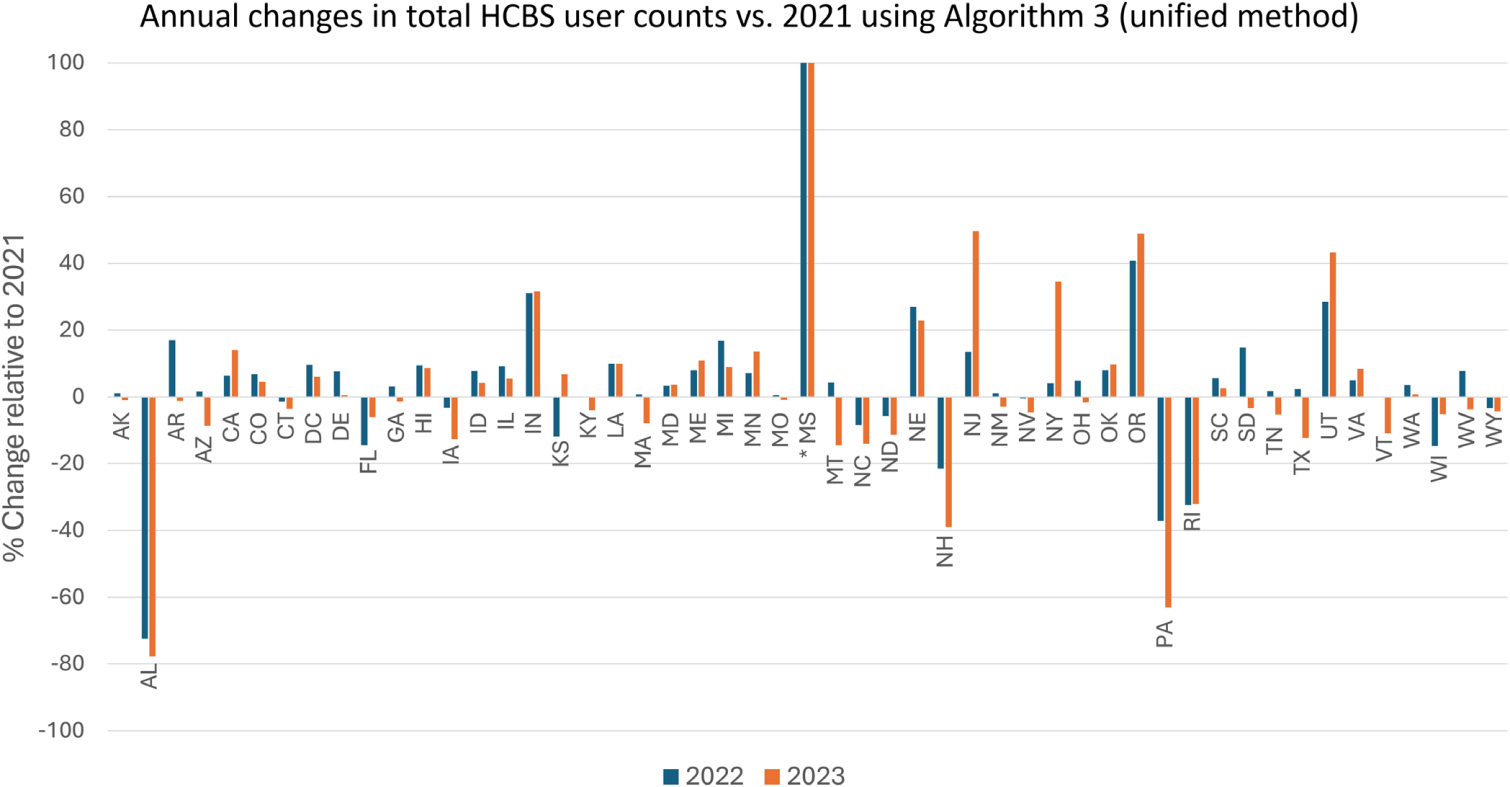
Year variations in HCBS user counts (per 100,000 residents) using the unified algorithm for 2022 and 2023, relative to 2021. For both 2022 and 2023, the DQ Atlas [20] reported that the 1915(c) waiver HCBS participant counts were unreliable (high concern) in NC, and unusable in MO and TN. *MS: The % change in Mississippi was truncated for reporting purposes in this graph because the actual % change was greater than 100% due to notable changes in data quality in 2022 (5293%) and 2023 (5253%).

Our assessment of state‐level time trends showed significant variations by year across states, suggesting data quality or reporting changes between 2021 and 2023 (Figure 3). For example, the implausibly high HCBS use counts for Pennsylvania in 2021 (approximately 1,300,000 HCBS users) were less elevated in 2022 and 2023. This trend might signal an improvement in the 2022 and 2023 data, relative to the elevated HCBS user counts in Pennsylvania for 2021. Similarly, implausibly low counts for Mississippi (less than 1000 HCBS users) in 2021 became more aligned with the state's enrollment patterns in the 2022 and 2023 data. In this case, the trend in HCBS users in Mississippi might indicate improved data reporting. The time trends for the unified approach are presented in Figure 3, and the time trends for the Mathematica method and Wang et al. method are shown in Figures S3 and S4, respectively.

To assess our 2021 sample data, we calculated the percent difference between our person‐year equivalent Medicaid enrollee counts and the benchmark person‐year equivalent enrollee counts from 2021 MACPAC data (Table S8). There was overall alignment between our enrollment counts and our benchmark counts, with a few exceptions. Arkansas, Illinois, Indiana, Missouri, and Tennessee all produced notably different counts (greater than 10% differences) when compared to the benchmark. Among these, Missouri and Tennessee were also noted as having data quality concerns in 2021 based on DQ Atlas and KFF assessments (Table S10).

Additionally, we calculated the number of enrollees in Medicaid 1915(c) waivers using the TAF D&E file, which we compared to enrollee counts reported by KFF and the DQ Atlas. Our TAF D&E file counts produced comparable counts to the benchmark counts (Table S9). There were no counts for Arizona, New Jersey, Rhode Island, and Vermont because those states do not provide HCBS services through 1915(c) waivers. Table S10 summarizes the state‐level data quality assessments for 2021, 2022, and 2023 based on our findings and state‐level data quality assessments by KFF and DQ Atlas.

## 4 Discussion

In this study, we compared two existing algorithms for identifying and classifying Medicaid HCBS claims in national TAF data. We also developed a unified algorithm that maintains the strengths of both approaches while introducing novel modifications to resolve disagreement between them. Using 2021 TAF data, we evaluated each algorithm's performance by constructing state‐level HCBS user counts. We also offer suggested approaches for assessing data quality for these services, including validating Waiver ID codes using the Medicaid.gov website, comparing 1915(c) waiver counts to KFF and DQ Atlas counts, measuring state‐level HCBS use per capita for comparability, and assessing year trends by state.

Overall, we noted a high level of agreement in the identification and classification of HCBS across algorithms. Among the cases of disagreement, we identified patterns that informed how we modified the unified approach. We found that the Mathematica approach (Algorithm 1) better identified state plan personal care services (Case 2), while Wang et al. (Algorithm 2) generally identified more state plan services overall (Case 4). Algorithm 1 also better identified capitated payments for waiver HCBS (Case 3) and captured waiver HCBS claims in Texas (Case 6). Algorithm 2 classified more ambiguous claims as waiver HCBS, including claims with a program type code indicating no special program (Case 7) and claims with program type 16 indicating 1915(j) state plan personal care services (Case 8). We verified these as waiver HCBS using 1915(c) waiver type codes and Waiver IDs.

Consistent with prior literature, we found state‐year variations in data quality and reporting patterns. In some states, there were inconsistencies in the HCBS users identified depending on the algorithm, while in others, all three algorithms yielded implausibly low or high counts. Differences in counts across algorithms suggest variation in how certain data elements are reported across states, underscoring trade‐offs in algorithm utility depending on which data elements are prioritized. In cases where all three algorithms yielded implausibly high or low counts, there may be systematic data quality concerns, which warrant ongoing monitoring. Furthermore, states and CMS should prioritize auditing the quality and consistency of state data submissions to TAF to enable program monitoring. Notably, the magnitudes of these implausible counts changed from 2021 to 2023, underscoring the need for researchers to remain attentive to potential state‐year‐level variations in data quality.

Our algorithm and analyses have several limitations. First, we do not evaluate specific categories of HCBS (e.g., personal care services, day services, nursing services) because of the unreliable quality of taxonomy variables, which differentiate service categories in the TAF data. Although some analysts have developed methods to map procedure codes to taxonomy categories, such methods are not feasible in cases where only Medicaid financing codes, but not procedure codes, are reported on HCBS claims (e.g., Case 3 in our analysis of discordant classifications). Second, benchmarking our findings was limited because, to our knowledge, there is no gold standard benchmark for HCBS user counts by state. Third, we had no way of tracking or quantifying the effects of the COVID‐19 pandemic‐era changes in HCBS user counts by state, which could reflect health policy changes during that time and temporary state‐specific reporting changes affecting 2021 data. Finally, our conclusions (based on algorithm performance) were subject to state‐ and year‐specific variations in data quality.

Despite these limitations, this study offers new insight into approaches for identifying and classifying HCBS that are beneficial for researchers. It also highlights cases where different algorithms produce substantially different estimates of HCBS users, highlighting inconsistencies that may reflect data concerns in specific states and years. These insights could also be useful for states and for CMS (Centers for Medicare and Medicaid Services) in their efforts to monitor and improve HCBS data reporting, a necessary aspect of program evaluation.

Our results also indicate the need for future work examining how the misclassification of certain types of HCBS could lead to systematic undercounting of HCBS use by particular demographic groups or individuals who disproportionately require certain services (e.g., personal care services). Additionally, these findings can inform further evidence generation on access to and use of Medicaid HCBS, which is needed to enhance quality and program integrity of Medicaid‐financed service provision.

## 5 Conclusion

Our findings emphasize that there is no perfect algorithm that can address all potential data quality or measurement issues across states, years, and types of HCBS being measured in the current version of TAF data. However, this work presents additional tools for identifying and classifying HCBS and outlines considerations that researchers should make when deciding on the best approach for their specific work. These considerations are critical in the measurement of HCBS usage because they support the development of evidence‐based research that can inform relevant Medicaid long‐term services and supports policy.

## Funding

This work was supported by Arnold Ventures, N/A.

## Conflicts of Interest

Ari Ne'eman, PhD, reports consulting income from the Service Employees International Union, the National Council on Disability, and CareSource. Dr. Ne'eman's views are his own and do not represent the perspective of these or any other entity. Kenton Johnston, PhD, reports funding from the NIH and Arnold Ventures outside of this work. Dr. Johnston also reports consulting fees from Brown University and honoraria from Project HOPE, and honoraria and support for travel from the American Academy of Neurology.

## Supporting information


**Table S1:** Description of TAF data elements used by each of the three algorithms for identifying and classifying HCBS.
**Table S2:** Description of Algorithm 1 (Mathematica method).
**Table S3:** Description of Algorithm 2 (Wang, Qi, and Konetzka method).
**Table S4:** Concordance calculations for Algorithm 1 and Algorithm 2.
**Table S5:** Description of Algorithm 3 (Unified, modified approach).
**Table S6:** List of TAF data elements and code descriptions used for Algorithm 3.
**Table S7:** Percentage of claims identified as state plan using program type code but 1915(c) waiver using waiver type codes (Algorithm 3, 2021 data).
**Table S8:** Benchmarking for 2021 data (TAF OT file): full‐year equivalent counts for continuously enrolled full‐benefit Medicaid beneficiaries aged 19+ in 2021, by state.
**Table S9:** Benchmarking for 2021 data (TAF D&E files): 1915(c) waiver HCBS user counts.
**Table S10:** State‐level data quality assessments for 2021, 2022, and 2023.
**Figure S1:** 1915(c) waiver HCBS users per 100,000 residents, by state in 2021, using three different approaches for identifying and classifying HCBS in the TAF OT files, and a fourth approach of obtaining waiver counts in the TAF DE file.
**Figure S2:** State plan HCBS (non‐waiver) users per 100,000 residents, by state, using three different approaches for identifying and classifying HCBS.
**Figure S3:** Year variations in HCBS user counts (per 100,000 residents) using the Mathematica method (Algorithm 1) for 2022 and 2023, relative to 2021.
**Figure S4:** Year variations in HCBS user counts (per 100,000 residents) using the Wang et al. method (Algorithm 2) for 2022 and 2023, relative to 2021.

## Data Availability

The data that support the findings of this study are available from Centers for Medicare & Medicaid Services. Restrictions apply to the availability of these data, which were used under license for this study. Data are available from the author(s) with the permission of Centers for Medicare & Medicaid Services.
