# Supplementary material for: How Should We Identify Home‐ and Community‐Based Services (HCBS) in Medicaid Claims Data? An Evaluation and Synthesis of Algorithms
Source: Health Serv Res. 2026 Sep 16;61(5):e70164. doi: 10.1111/1475-6773.70164 (PMC13583170; doi:10.1111/1475-6773.70164)
Supplement: Supplementary file 1 [file HESR-61-e70164-s001.docx]

**APPENDIX**

Appendix Table 1. Description of TAF data elements used by each of the three algorithms for identifying and classifying HCBS

| TAF Data elements | | | Algorithm 1  (Mathematica) | Algorithm 2  (Wang et al.) | Algorithm 3  (Unified) |
| --- | --- | --- | --- | --- | --- |
|  | **Codes** | **Description** ^a^ |  |  |  |
| Program-related codes | Program type (*PGM_TYPE_CD*) | The type of Medicaid (or CHIP) program that a particular claim is associated with. | Yes | Yes | Yes |
|  | Waiver type  *(WVR_TYPE_CD)* | The waiver type under which the eligible beneficiary is covered and receiving services during the month, or under which a claim is submitted. | Yes | Yes | Yes |
|  | Waiver identifiers  *(WVR_ID)* | The ID number for the waiver which authorized payment for a claim. | No | No | Yes |
| Service-related codes | Type of service  *(TOS_CD)* | Categorizes the services provided to a Medicaid or CHIP enrollee. | Yes | Yes | Yes |
|  | Benefit type  *(BNFT_TYPE_CD)* | Specifies the benefit category corresponding to the service reported on the claim or encounter record. | Yes | No | Yes |
|  | HCBS taxonomy  *(HCBS_TXNMY_CD)* | Classifies HCBS listed on the claim into the HCBS taxonomy. | No | Yes | No |
|  | HCBS service  *(HCBS_SRVC_CD)* | The authority under which HCBS is provided for a particular claim, for example: 1915(c) waiver or 1915 (i), (j), or (k). | Yes | No | Yes |
| Care delivery or billing codes | Place of service  *(POS_CD)* | Where the service for a particular claim was performed. | No | Yes | Yes |
|  | Procedure  *(LINE_PRCDR_CD)* | The CPT or HCPCS code that describes a service or good rendered by the provider to an enrollee on the specified date of service. | Yes | Yes | Yes |

*a. As described in the T-MSIS Analytic Files (TAF) Claims Research Identifiable Files (RIFs) Codebook, Version 1.7. Published online April 2025.*

Appendix Table 2. Description of Algorithm 1 (Mathematica method)

| **Algorithm 1. Mathematica method** | | | |
| --- | --- | --- | --- |
| *Identifying 1915(c) HCBS* | *Identifying state plan HCBS* | | |
| 1915(c) waiver:   1. Program type code= 07 2. If Program type code missing: Waiver type code = 06 - 20, 33. 3. If Program type code and Waiver type code missing: HCBS service code = 4. | 1915 (i) (j) and (k):   1. Program type code = 11, 13, or 16. 2. If program type code and waiver type code missing: HCBS service code = 1, 2 or 3. 3. If program type, waiver type, and HCBS service code missing: Benefit type code = 054 or 106. | State plan personal care services:  Claim is not identified as a program-based claim and procedure code = T1019, T1020, 99509, S5125, or S5126. | MFP demonstration and  all other HCBS:   1. Program type code= 08. 2. If claim is not identified as a program-based claim: TOS = 016 - 022, 043, 053, 054, 062, 064, 077, or 079. 3. If claim is not identified as a program-based claim and TOS code missing: Benefit type code= 015, 016, 017, 022, 023, 036, 042, 068, 069, or 076. |

See Appendix Table 1 for descriptions of the variables used in this algorithm.

Appendix Table 3. Description of Algorithm 2 (Wang, Qi, and Konetzka method)

| **Algorithm 2. Wang, Qi, and Konetzka method** | | | |
| --- | --- | --- | --- |
| *Identifying 1915(c) HCBS* | | | *Identifying state plan HCBS* |
| Type of service approach   - TOS = 016 - 021, 036, 043, 051, 053, 054, 062 - 083, 115. - or POS = 12 and TOS = 022, 087. | HCBS taxonomy approach*  All valid 5-digit values | Procedure code approach  Procedure codes mapped to HCBS taxonomy crosswalk * | Type of service approach   - TOS = 016 - 021, 036, 043, 051, 053, 054, 062 - 083, 115 - or POS = 12 and TOS = 022, 087. |
| **And**   - Program type code= 07 - or Waiver type code = 06 - 20, 33. | | | **and**  Program type code != 07 |
|  |  |  | **and**  Waiver type code != 06 - 20, 33. |

See Appendix Table 1 for descriptions of the variables used in this algorithm.**The authors use a crosswalk of HCBS taxonomy codes and procedure codes, constructed using legacy MAX data, to obtain a full list of procedure codes for identifying HCBS.*

Appendix Table 4. Concordance calculations for Algorithm 1 and Algorithm 2

| *Description* | *Calculation** |
| --- | --- |
| **Observed agreement,** $p_{o}:$  sum of diagonal proportions (Total %) in Table 1. | $p_{o}$= (0.80 + 0.07 + 0.06) = 0.93 |
| **Expected agreement,** $p_{e}$ :  Multiply each category’s row % total (from Algorithm 2) by its corresponding column % total (from Algorithm 1) and sum the products across all categories. | $p_{e}$ = (0.82 x 0.83) + (0.09 x 0.10) + (0.09 + 0.07) = 0.696 |
| **Cohen’s Kappa statistic,** $\kappa$ **:**  $\kappa= \left( p_{o}- p_{e} \right) / (1 -p_{e})$ | $\kappa$ = (0.93 – 0.696) / (1 – 0.696) = **0.77** |

** based on Table 1 results, comparing algorithm performance for Algorithm 1 and Algorithm 2*

Appendix Table 5. Description of Algorithm 3 (Unified, modified approach)

| **Algorithm 3. Modified, unified approach** | |
| --- | --- |
| *Identifying 1915(c) HCBS* | *Identifying state plan HCBS* |
| Any of the following criteria:   - Program type code = 07. - Program type code = 16 (with 1915(c) Waiver ID code). - Waiver type code = 06 - 20, 33. - Missing program type and waiver type code and HCBS service code = 4. | Any of the following criteria:   - Program type code = 08, 11, 13 - Program type code = 16 (without 1915(c) waiver ID) - Program type code is 00 or missing and: - Procedure code = T1019, T1020, 99509, S5125, S5126 - TOS = 016 - 021, 036, 043, 051, 053, 054, 062- 083, 115 or - POS = 12 and TOS = 022 or - Missing TOS code and Benefit type code= 015, 016, 017, 022, 023, 036, 042, 068, 069, 076 - Missing program type and waiver type code and HCBS service code = 1, 2, 3. - Missing program type, waiver type, and HCBS service code and benefit type code = 054, 106. |
|  | **and**   - Program type code != 07, - and Program type code!= 16 with 1915(c) waiver ID, - and Waiver type code != 06 - 20, 33 - and not (missing program type code and waiver type code and HCBS service code = 4). |

See Appendix Table 1 for description of the variables used in this algorithm.

Appendix Table 6. List of TAF data elements and code descriptions used for Algorithm 3

| **TAF Data element** |  | **Codes and descriptions** |
| --- | --- | --- |
| Program type code^20^ | 00 | No Special Program |
|  | 07 | Home and Community-Based Services (HCBS) |
|  | 08 | Money Follows the Person (MFP) |
|  | 11 | Community First Choice (1915(k)) |
|  | 13 | HCBS State Plan Option (1915(i)) |
|  | 16 | 1915(j) (Self- directed personal assistance services/personal care under State Plan or 1915(c) waiver) |
| Waiver type code^21^ | 06 | 1915(c) Aged and Disabled |
|  | 07 | 1915(c) Aged |
|  | 08 | 1915(c) Physical Disabilities |
|  | 09 | 1915(c) Intellectual Disabilities |
|  | 10 | 1915(c) Intellectual and Developmental Disabilities |
|  | 11 | 1915(c) Brain Injury |
|  | 12 | 1915(c) HIV/AIDS |
|  | 13 | 1915(c) Technology Dependent or Medically Fragile |
|  | 14 | 1915(c) Disabled (other) |
|  | 15 | 1915(c) Enrolled in 1915(c) waiver for unspecified or unknown populations |
|  | 16 | 1915(c) Autism/Autism Spectrum Disorder |
|  | 17 | 1915(c) Developmental Disabilities |
|  | 18 | 1915(c) Mental Illness, age 18 or older |
|  | 19 | 1915(c) Mental Illness, under age 18 |
|  | 20 | 1915(c) waiver concurrent with an 1115 or 1915(b) managed care authority |
|  | 33 | 1915(c) waiver |
| Waiver ID codes^18^ | Waiver IDs for 1915(c) HCBS were identified using their unique format XX.####, where XX indicates the state name abbreviation and #### is the unique number associated with that specific waiver ID. | |
|  | DE.0009 | Delaware, Delaware Division of Developmental Disabilities (DDDS) Lifespan Waiver |
|  | ID.1076 | Idaho, Aged and Disabled waiver |
|  | IN.02160 | Indiana *(identified based on format)* |
|  | IN.037805 | Indiana, Community Integration and Habilitation Waiver |
|  | IN.03873 | Indiana *(identified based on format)* |
|  | IN.41970400 | Indiana *(identified based on format)* |
|  | KY.0144 | Kentucky, Home and Community Based Waiver |
|  | KY.0314 | Kentucky, Supports for Community Living Waiver |
|  | KY.0333 | Kentucky, Acquired Brain Injury Waiver |
|  | KY.0475 | Kentucky, Michelle P. Waiver |
|  | KY.0477 | Kentucky, Acquired Brain Injury – Long Term Care Waiver |
|  | MA.0059 | Massachusetts, Frail Elder Waiver |
|  | MA.1027 | Massachusetts, MFP Community Living (MFP – CL) Waiver |
|  | MA.40702 | Massachusetts, Acquired Brain Injury Non-Residential Habilitation (ABI – N) Waiver |
|  | NC.0423 | North Carolina, Innovations Waiver |
|  | NM.1726 | New Mexico, Supports Waiver |
|  | NM.448 | New Mexico, Mi Via - ICF/IDD Waiver |
|  | NV.0125 | Nevada, Waiver for individuals with IDD |
|  | NV.0152 | Nevada, Waiver for the Frail Elderly |
|  | NV.4150 | Nevada, HCBS Waiver for Persons with Physical Disabilities |
|  | NY.0238 | New York, OPWDD Comprehensive Waiver |
|  | NY.0269 | New York, Traumatic Brain Injury (TBI) Waiver |
|  | NY.0444 | New York, Nursing Home Transition and Diversion Medicaid Waiver |
|  | OH.0198 | Ohio, PASSPORT waiver |
|  | OH.0231 | Ohio, Individual Options Waiver |
|  | OH.0337 | Ohio, Home Care Waiver |
|  | OH.0380 | Ohio, Level One Waiver |
|  | OH.0877 | Ohio, Self Empowered Life Funding (SELF) Waiver |
|  | OK.0256 | Oklahoma, Advantage waiver |
|  | OK.0811 | Oklahoma, Medically Fragile Waiver |
|  | PA.0147 | Pennsylvania, Consolidated waiver for individuals with IDD |
|  | PA.0235 | Pennsylvania, OBRA waiver for individuals with IDD |
|  | PA.0354 | Pennsylvania, Person/Family Directed Support waiver for individuals IDD |
|  | PA.0593 | Pennsylvania, Adult Autism Waiver (AAW) |
|  | PA.1486 | Pennsylvania, Community Living waiver |
|  | TX.0110 | Texas, IDD waiver |
|  | TX.0221 | Texas, Community Living Assistance and Support Services (CLASS) waiver |
|  | TX.0281 | Texas, Deaf Blind with Multiple Disabilities waiver |
|  | TX.0403 | Texas, Home Living waiver |
|  | WA.0049 | Washington, Community Options Program Entry System (COPES) waiver |
|  | WA.0443 | Washington, New Freedom waiver |
|  | WA.1086 | Washington, Residential Support waiver |
|  | WI.0367 | Wisconsin, Family Care waiver |
|  | WI.0484 | Wisconsin, Include, Respect, I Self-Direct (IRIS) waiver |
|  | WV.0133 | West Virginia, IDD waiver |
|  | WV.0134 | West Virginia, Aged and Disabled waiver |
|  | WV.0876 | West Virginia, Traumatic Brain Injury (TBI) waiver |
| Type of service code^22^ | 016 | Home health services: Nursing services |
|  | 017 | Home health services: Home health aide services |
|  | 018 | Home health services: Medical supplies, equipment, and appliances |
|  | 019 | Home health services: Physical therapy |
|  | 020 | Home health services: Occupational therapy |
|  | 021 | Home health services: Speech pathology and audiology services |
|  | 022 | Private duty nursing services |
|  | 036 | Medical equipment/prosthetic devices |
|  | 043 | Rehabilitative services |
|  | 051 | Personal care services |
|  | 053 | Targeted case management services |
|  | 054 | Case Management services, other (not primary care case management services or targeted case management services) |
|  | 062 | HCBS: Case management services |
|  | 063 | HCBS: Homemaker services |
|  | 064 | HCBS: Home health aide services |
|  | 065 | HCBS: Personal care services |
|  | 066 | HCBS: Adult day health services |
|  | 067 | HCBS: Habilitation services |
|  | 068 | HCBS: Respite care services |
|  | 069 | HCBS: Day treatment or other partial hospitalization services, psychosocial  rehabilitation services and clinic services for individuals with chronic mental illness |
|  | 070 | HCBS: Day Care |
|  | 071 | HCBS: Training for family members |
|  | 072 | HCBS: Minor modification to the home |
|  | 073 | HCBS: Other services requested by the agency and approved by CMS as cost  effective and necessary to avoid institutionalization |
|  | 074 | HCBS: Expanded habilitation services (Prevocational services) |
|  | 075 | HCBS: Expanded habilitation services (Educational services) |
|  | 076 | HCBS: Expanded habilitation services (Supported employment services which  facilitate paid employment) |
|  | 077 | HCBS, 65 plus: Case management services |
|  | 078 | HCBS, 65 plus: Homemaker services |
|  | 079 | HCBS, 65 plus: Home health aide services |
|  | 080 | HCBS, 65 plus: Personal care services |
|  | 081 | HCBS, 65 plus: Adult day health services |
|  | 082 | HCBS, 65 plus: Respite care services |
|  | 083 | HCBS, 65 plus: Other medical and social services |
|  | 115 | Residential care |
| Benefit type code^23^ | 015 | Home Health Services: Intermittent or part-time nursing services provided by a home health agency |
|  | 016 | Home Health Services: Home Health Aide Services provided by a home health agency |
|  | 017 | Home Health Services: Medical supplies, equipment, and appliances suitable for use in the home |
|  | 022 | Home Health Services: Physical therapy; occupational therapy; speech pathology; audiology provided by a home health agency |
|  | 023 | Private Duty Nursing |
|  | 036 | Other diagnostic, screening, preventive, and rehabilitative services |
|  | 042 | Case Management Services and TB related services: Case management services as defined in the State Plan in accordance with section 1905(a)(19) or 1915(g) |
|  | 054 | Community First Choice |
|  | 068 | Home Health Services: Home health aide services provided by a home health agency |
|  | 069 | Private duty nursing services |
|  | 076 | Home Health Aide |
|  | 106 | Self-directed Personal Assistance Services under 1915(j) |
| HCBS service code^24^ | 1 | HCBS service was provided under 1915(i) |
|  | 2 | HCBS service was provided under 1915(j) |
|  | 3 | HCBS service was provided under 1915(k) |
|  | 4 | HCBS service was provided under a 1915(c) HCBS Waiver |
| Place of service code^25^ | 12 | Home. Location, other than a hospital or other facility, where the patient receives care in a private residence. |
| Procedure code | T1019 | Personal care services in a non-institutional setting (15 Minutes = 1 Unit) |
|  | T1020 | Personal Care Services, per diem (8 hrs.) |
|  | 99509 | Home visit where a provider helps with activities of daily living |
|  | S5125 | Self-directed attendant care service (15 Minutes = 1 unit) |
|  | S5126 | Attendant care services, per diem (8hrs.) |
|  |  |  |

Appendix Table 7**.** Percentage of claims identified as state plan using program type code but 1915(c) waiver using waiver type codes (Algorithm 3, 2021 data).

| **PGM_TYPE_CD** | **% with a waiver type code of 06-20, 33** |
| --- | --- |
| 08, 11, or 13 | 1.4% |
| Other (i.e. not 00, 07, 08, 11, 13, or 16) | 0.6% |
| 00 | 2.2% |
| Missing | 0.6% |

Appendix Table 8. Benchmarking for 2021 data (TAF OT file): full-year equivalent counts for continuously enrolled full-benefit Medicaid beneficiaries aged 19+ in 2021, by state

| State | | Full-year  Equivalent  OT file counts ^a^ | Benchmark  Data: MACPAC ^b^ | % difference | |
| --- | --- | --- | --- | --- | --- |
|  |  |  |  | (relative to our OT  file counts) | Data quality concern (>10%) ^c^ |
| AK | Alaska | 148,354 | 141,737 | 4.7 | No |
| AL | Alabama | 344,708 | 341,482 | 0.9 | No |
| AR | Arkansas | 536,587 | 472,535 | 13.6 | Yes |
| AZ | Arizona | 1,219,572 | 1,198,914 | 1.7 | No |
| CA | California | 8,185,757 | 7,800,158 | 4.9 | No |
| CO | Colorado | 911,821 | 884,611 | 3.1 | No |
| CT | Connecticut | 627,575 | 600,919 | 4.4 | No |
| DC | District of Columbia | 181,061 | 178,113 | 1.7 | No |
| DE | Delaware | 146,740 | 141,804 | 3.5 | No |
| FL | Florida | 1,786,594 | 1,797,515 | -0.6 | No |
| GA | Georgia | 737,484 | 713,637 | 3.3 | No |
| HI | Hawaii | 265,867 | 256,009 | 3.9 | No |
| IA | Iowa | 424,798 | 431,262 | -1.5 | No |
| ID | Idaho | 204,114 | 205,314 | -0.6 | No |
| IL | Illinois | 1,905,600 | 2,333,563 | -18.3 | Yes |
| IN | Indiana | 1,043,528 | 937,016 | 11.4 | Yes |
| KS | Kansas | 158,493 | 152,911 | 3.7 | No |
| KY | Kentucky | 1,008,287 | 1,055,190 | -4.4 | No |
| LA | Louisiana | 1,009,241 | 1,016,421 | -0.7 | No |
| MA | Massachusetts | 1,191,496 | 1,157,162 | 3.0 | No |
| MD | Maryland | 872,441 | 816,793 | 6.8 | No |
| ME | Maine | 210,340 | 204,866 | 2.7 | No |
| MI | Michigan | 1,759,225 | 1,751,244 | 0.5 | No |
| MN | Minnesota | 688,702 | 636,076 | 8.3 | No |
| MO | Missouri | 496,376 | 439,856 | 12.8 | Yes |
| MS | Mississippi | 233,077 | 255,562 | -8.8 | No |
| MT | Montana | 167,122 | 158,126 | 5.7 | No |
| NC | North Carolina | 834,588 | 837,561 | -0.4 | No |
| ND | North Dakota | 62,645 | 60,404 | 3.7 | No |
| NE | Nebraska | 149,635 | 140,594 | 6.4 | No |
| NH | New Hampshire | 130,839 | 124,286 | 5.3 | No |
| NJ | New Jersey | 1,045,439 | 1,133,398 | -7.8 | No |
| NM | New Mexico | 499,125 | 484,455 | 3.0 | No |
| NV | Nevada | 438,127 | 438,164 | 0.0 | No |
| NY | New York | 4,334,672 | 4,684,759 | -7.5 | No |
| OH | Ohio | 1,828,161 | 1,761,255 | 3.8 | No |
| OK | Oklahoma | 354,762 | 354,632 | 0.0 | No |
| OR | Oregon | 773,209 | 718,080 | 7.7 | No |
| PA | Pennsylvania | 1,983,220 | 2,053,114 | -3.4 | No |
| RI | Rhode Island | 218,365 | 214,062 | 2.0 | No |
| SC | South Carolina | 514,447 | 471,222 | 9.2 | No |
| SD | South Dakota | 42,860 | 42,851 | 0.0 | No |
| TN | Tennessee | 944,928 | 702,960 | 34.4 | Yes |
| TX | Texas | 1,428,512 | 1,303,717 | 9.6 | No |
| UT | Utah | 207,176 | 199,743 | 3.7 | No |
| VA | Virginia | 988,474 | 985,993 | 0.3 | No |
| VT | Vermont | 113,771 | 112,033 | 1.6 | No |
| WA | Washington | 1,142,937 | 1,112,149 | 2.8 | No |
| WI | Wisconsin | 746,332 | 772,147 | -3.3 | No |
| WV | West Virginia | 361,941 | 353,707 | 2.3 | No |
| WY | Wyoming | 26,215 | 25,268 | 3.7 | No |

*This table compares counts of full-benefit Medicaid enrollees ages 19 and older from our analysis of the TAF DE file with counts reported in MACStats data. The purpose was to check whether the population of Medicaid enrollees, among whom we then measured HCBS use, was comparable what MACStats reported, using MACStats as a benchmark.*

*a.* ***Full-year Equivalent OT file counts****. Medicaid Enrollee counts calculated for our sample using the CMS definition: N months where age>=19 and RSTRCTD_BNFT_CD= 1,7,D in 2021 divided by 12*

*b.* ***Benchmark Data: MACPAC.*** *MACPAC data from December 2023 MACStats data, Exhibit 15. Medicaid full-year equivalent enrollment, FY2021*

*c.* ***Data quality concern (>10%).*** *The 10% data quality threshold aligns with the DQ Atlas’ “low concern” rating for data that deviates from the benchmark by 10% or less. In this case, our benchmark is the MACPAC data for full-year equivalent enrollment in 2021.*

Appendix Table 9**.** Benchmarking for 2021 data (TAF D&E files): 1915(c) waiver HCBS user counts

| State | | One month or more of 1915(c) waiver | | | 1915c waiver type code flag | | |
| --- | --- | --- | --- | --- | --- | --- | --- |
|  |  | Enrollees with > one month of 1915 (c) enrollment | KFF  1915 (c) waiver counts ^a^ | % difference | Enrollees covered under the 1915(c) waiver | DQ Atlas  1915(c)  waiver  counts ^b^ | % difference |
| AK | Alaska | 5,128 | 5,100 | 0.5 | 5,128 | 5,127 | 0.0 |
| AL | Alabama | 17,146 | 17,100 | 0.3 | 34,760 | 34,760 | 0.0 |
| AR | Arkansas | 9,765 | 9,800 | -0.4 | 9,765 | 9,765 | 0.0 |
| AZ | Arizona | 0 | 0 | NA | 0 | NA | NA |
| CA | California | 170,108 | 164,300 | 3.5 | 170,108 | 164,328 | 3.5 |
| CO | Colorado | 59,404 | 59,400 | 0.0 | 59,404 | 59,403 | 0.0 |
| CT | Connecticut | 30,950 | 31,000 | -0.2 | 30,950 | 30,950 | 0.0 |
| DC | District of Columbia | 7,645 | 7,600 | 0.6 | 7,645 | 7,645 | 0.0 |
| DE | Delaware | 1,694 | 1,700 | -0.4 | 1,694 | 1,694 | 0.0 |
| FL | Florida | 4,715 | 4,700 | 0.3 | 141,667 | Suppressed | NA |
| GA | Georgia | 57,715 | 57,700 | 0.0 | 57,715 | 57,704 | 0.0 |
| HI | Hawaii | 3,204 | 3,200 | 0.1 | 3,204 | 3,173 | 1.0 |
| IA | Iowa | 29,245 | 29,200 | 0.2 | 29,245 | 29,245 | 0.0 |
| ID | Idaho | 23,599 | 23,500 | 0.4 | 23,599 | 23,522 | 0.3 |
| IL | Illinois | 178,189 | 178,000 | 0.1 | 178,189 | 178,043 | 0.1 |
| IN | Indiana | 76,155 | 75,300 | 1.1 | 76,155 | 75,328 | 1.1 |
| KS | Kansas | 0 | 0 | NA | 30,763 | 30,757 | 0.0 |
| KY | Kentucky | 29,661 | 29,600 | 0.2 | 29,661 | 29,555 | 0.4 |
| LA | Louisiana | 20,329 | 20,300 | 0.1 | 24,144 | 23,864 | 1.2 |
| MA | Massachusetts | 30,967 | 31,000 | -0.1 | 30,967 | 30,958 | 0.0 |
| MD | Maryland | 27,282 | 27,300 | -0.1 | 27,282 | 27,261 | 0.1 |
| ME | Maine | 0 | 0 | NA | 7,253 | 7,253 | 0.0 |
| MI | Michigan | 9,134 | 9,100 | 0.4 | 31,314 | 31,298 | 0.1 |
| MN | Minnesota | 92,214 | 92,200 | 0.0 | 96,967 | 96,967 | 0.0 |
| MO | Missouri | 0 | 0 | NA | 0 | 0 | NA |
| MS | Mississippi | 1,248 | DQ* | NA | 1,248 | 1,248 | 0.0 |
| MT | Montana | 5,790 | 5,800 | -0.2 | 5,790 | 5,788 | 0.0 |
| NC | North Carolina | 0 | 0 | NA | 1,892,100 | 1,745,946 | 8.4 |
| ND | North Dakota | 6,490 | 6,500 | -0.2 | 6,490 | 6,485 | 0.1 |
| NE | Nebraska | 39 | NSD | NA | 12,495 | 12,495 | 0.0 |
| NH | New Hampshire | 10,909 | 10,800 | 1.0 | 10,909 | 10,843 | 0.6 |
| NJ | New Jersey | 12,427 | 12,400 | 0.2 | 12,427 | 12,427 | 0.0 |
| NM | New Mexico | 5,593 | 5,600 | -0.1 | 5,593 | 5,592 | 0.0 |
| NV | Nevada | 7,773 | 7,800 | -0.3 | 7,773 | 7,773 | 0.0 |
| NY | New York | 131,287 | 130,800 | 0.4 | 131,287 | 130,829 | 0.4 |
| OH | Ohio | 84,350 | 84,200 | 0.2 | 118,836 | 118,667 | 0.1 |
| OK | Oklahoma | 28,258 | 28,300 | -0.1 | 28,258 | 28,258 | 0.0 |
| OR | Oregon | 34,835 | 34,800 | 0.1 | 34,835 | 34,789 | 0.1 |
| PA | Pennsylvania | 33,611 | 33,600 | 0.0 | 129,097 | 129,097 | 0.0 |
| RI | Rhode Island | 0 | 0 | NA | 0 | NA | NA |
| SC | South Carolina | 46,378 | 46,200 | 0.4 | 46,378 | 46,240 | 0.3 |
| SD | South Dakota | 5,779 | 5,800 | -0.4 | 5,779 | 5,777 | 0.0 |
| TN | Tennessee | 0 | 0 | NA | 0 | 0 | NA |
| TX | Texas | 43,745 | 43,700 | 0.1 | 50,042 | 49,941 | 0.2 |
| UT | Utah | 8,850 | 8,900 | -0.6 | 8,850 | 8,850 | 0.0 |
| VA | Virginia | 63,586 | 63,500 | 0.1 | 63,586 | 63,538 | 0.1 |
| VT | Vermont | 0 | 0 | NA | 0 | NA | NA |
| WA | Washington | 53,660 | 53,700 | -0.1 | 53,660 | 53,660 | 0.0 |
| WI | Wisconsin | 25,666 | 25,700 | -0.1 | 104,421 | 104,193 | 0.2 |
| WV | West Virginia | 17,833 | 17,800 | 0.2 | 17,833 | 17,815 | 0.1 |
| WY | Wyoming | 5,487 | 5,500 | -0.2 | 5,487 | 5,487 | 0.0 |

*This table compares our counts of full-benefit Medicaid beneficiaries enrolled for at least one month in a 1915(c) HCBS waiver in 2021, derived from the TAF DE file, with counts reported by KFF (left columns) and the Medicaid DQ Atlas (right columns). The purpose was to check that waiver enrollment estimates from our analysis of the TAF DE file are consistent with previously published counts using these data sources.*

a. ***KFF section 1915(c) waiver counts****. Enrollees who had at least one month of 1915(c) enrollment (WVR_1915C_MOS > 0) were identified as using 1915(c) waiver services.*

*b.* ***DQ Atlas 1915(c) participant count in TAF DE file****. Calculated using the 1915(c) waiver flag (_1915C_WVR_TYPE).*

* State flagged as having unusable data in KFF analysis

Appendix Table 10 . State-level data quality assessments for 2021, 2022, and 2023

|  | **2021** | **2022** | **2023** |
| --- | --- | --- | --- |
| **Assessing comparability of**  **Medicaid enrollment counts** |  |  |  |
| DQ Atlas assessment: concerns with one or more variables needed to identify full Medicaid enrollees. ^a^ | DC, IA, KY,  MS, NJ, TN | DC, IA,  RI, VT | DC, IA |
| Our assessment: high % differences (>10%) between OT file and MACPAC counts (*see Appendix Table 6*). | AR, IL, IN, MO, TN |  |  |
| **Assessing the data quality of**  **1915(c) HCBS waiver counts** |  |  |  |
| States without 1915(c) waivers for HCBS. | AZ, NJ, RI, VT | AZ, NJ, RI, VT | AZ, NJ, RI, VT |
| DQ Atlas assessment: data quality concerns with 1915(c) participant data. | FL, MO, TN | FL, MO, MS,  NC, PA, TN | MO, NC, PA,  SD, TN |
| KFF: States with missing or suppressed data for 1915(c) counts . | KS, ME, MS,  MO, NE, NC, TN | KS, ME, MI,  MO, NC, TN | KS, ME, MI,  MO, NC, TN |
| Our assessment: high % differences (>10%) between DE file and DQ Atlas or KFF counts (*see Appendix Table 7*). | (None) |  |  |

a. ***DQ Atlas state-level assessment.*** *States noted for high concern, or unusable data based on the quality TAF data (relative to Performance Indicator data), service use records that can be linked to eligibility records, or the quality of eligibility group code and restricted benefits code data.*

Appendix Figure 1. 1915(c) waiver HCBS users per 100,000 residents, by state in 2021, using three different approaches for identifying and classifying HCBS in the TAF OT files, and a fourth approach of obtaining waiver counts in the TAF DE file.

*Note: The DQ Atlas did not report assessments of the reliability of data for 1915(c) waiver HCBS participation by state in 2021*^15^*.*

*Most states show agreement across approaches. Seven states have general agreement between algorithms, but lower counts (AL, DE, MO, NJ, TN, VT, and WA) or higher counts (FL) based on the DE waiver file. SD, PA, and IL show variation in counts across algorithms and the DE waiver file.*

Appendix Figure 2. State plan HCBS (non-waiver) users per 100,000 residents, by state, using three different approaches for identifying and classifying HCBS

State plan HCBS users per 100,000 residents, based on three HCBS identification methods

|  | Wang et al. | Mathematica | Unified |
| --- | --- | --- | --- |
| AK | 2342.54 | 1362.85 | 2448.22 |
| AL | 409.511 | 589.935 | 410.805 |
| AR | 1831.46 | 738.663 | 1836.94 |
| AZ | 1406.84 | 1369.01 | 1399.85 |
| CA | 2728.9 | 408.339 | 2714.39 |
| CO | 1820.84 | 754.194 | 1825.51 |
| CT | 2387.72 | 1238.65 | 2410.19 |
| DC | 6131.58 | 1288.53 | 5763.22 |
| DE | 2048.59 | 1155.62 | 2049.1 |
| FL | 842.132 | 545.218 | 867.668 |
| GA | 707.857 | 644.563 | 707.344 |
| HI | 1656.74 | 1648.49 | 1649.52 |
| IA | 740.04 | 753.518 | 576.266 |
| ID | 436.353 | 491.543 | 436.625 |
| IL | 1851.08 | 937.912 | 1851.04 |
| IN | 1292.53 | 665.46 | 1293.71 |
| KS | 1830.23 | 756.498 | 1828.36 |
| KY | 3325.02 | 482.707 | 3324.8 |
| LA | 1761.04 | 720.626 | 1713.7 |
| MA | 3647.48 | 1431.95 | 2524.5 |
| MD | 1911.38 | 1693.16 | 1979.69 |
| ME | 633.093 | 666.858 | 651.811 |
| MI | 3518.49 | 2124.02 | 3429.68 |
| MN | 4120.39 | 1354.42 | 4117.68 |
| MO | 1821.72 | 1345.84 | 1822.12 |
| MS | 5.40307 | 2.02615 | 5.40307 |
| MT | 2469.97 | 518.804 | 2469.97 |
| NC | 1259.94 | 811.063 | 1259.84 |
| ND | 1881.03 | 709.799 | 1875.26 |
| NE | 5947.22 | 5911.79 | 5912.55 |
| NH | 1018.85 | 1010.87 | 1019.22 |
| NJ | 1666 | 457.046 | 1665.3 |
| NM | 3525.35 | 3350.14 | 3525.11 |
| NV | 1048.6 | 521.16 | 1048.76 |
| NY | 3306.9 | 3339.03 | 3308.41 |
| OH | 4226 | 3282.99 | 4207.99 |
| OK | 322.654 | 619.798 | 313.814 |
| OR | 6291.81 | 6280.83 | 6290.98 |
| PA | 2798.19 | 3427.37 | 2796.96 |
| RI | 2385.68 | 2486.38 | 2497.31 |
| SC | 1321.4 | 662.508 | 1313.2 |
| SD | 969.924 | 312.406 | 955.827 |
| TN | 1633.67 | 411.672 | 1627.01 |
| TX | 981.541 | 1224.63 | 1244.94 |
| UT | 83.445 | 73.8167 | 79.6243 |
| VA | 934.612 | 681.929 | 935.596 |
| VT | 2615.55 | 645.335 | 2614.77 |
| WA | 2525.48 | 1160.5 | 2502.12 |
| WI | 2744.28 | 1917.47 | 2740.55 |
| WV | 4266.56 | 4161.47 | 4255.41 |
| WY | 210.626 | 165.554 | 208.026 |

*Note: The DQ Atlas did not report assessments of the reliability of data for 1915(c) waiver HCBS participation by state in 2021*^15^*.*

Appendix Figure 3. Year variations in HCBS user counts (per 100,000 residents) using the Mathematica method (Algorithm 1) for 2022 and 2023, relative to 2021

*

Annual changes in total HCBS user counts vs. 2021 using Algorithm 1 (Mathematica method)

% Change relative to 2021

|  | 2022 | 2023 |
| --- | --- | --- |
| AK | 1.02954 | 0.358102 |
| AL | -77.8576 | -81.0898 |
| AR | 22.9657 | 14.0764 |
| AZ | 1.8229 | -8.53913 |
| CA | 11.7576 | 44.631 |
| CO | -4.78392 | -5.82755 |
| CT | -1.83338 | -2.21336 |
| DC | 18.3199 | 9.29401 |
| DE | 8.11442 | 1.95563 |
| FL | -25.1625 | -11.169 |
| GA | 3.38877 | -0.891781 |
| HI | 9.23164 | 8.4765 |
| IA | -3.04594 | -12.0579 |
| ID | 7.48212 | 1.762 |
| IL | 9.10934 | 5.48883 |
| IN | 28.3408 | 39.2194 |
| KS | 3.4162 | 53.7288 |
| KY | -2.04611 | -7.46679 |
| LA | -1.82898 | 6.90024 |
| MA | -3.47922 | -14.1259 |
| MD | 3.49452 | 3.00812 |
| ME | 7.92242 | 12.8534 |
| MI | 3.72912 | -2.14374 |
| MN | 3.27015 | 9.28937 |
| MO | 1.92399 | 2.51006 |
| MS | 6990.12 | 6669.14 |
| MT | -0.92091 | -5.6338 |
| NC | -1.95125 | 18.9395 |
| ND | -12.3596 | -19.8942 |
| NE | 26.9625 | 22.5489 |
| NH | -21.559 | -39.1047 |
| NJ | 6.9626 | 109.324 |
| NM | 0.603608 | -3.53934 |
| NV | 3.39623 | 0.301887 |
| NY | 4.06646 | 34.2212 |
| OH | 2.33076 | -6.20971 |
| OK | 8.87719 | 12.1579 |
| OR | 41.8261 | 49.8481 |
| PA | 6.42836 | -38.2371 |
| RI | -32.2161 | -31.7216 |
| SC | -1.90619 | -8.06026 |
| SD | 4.77915 | -2.67922 |
| TN | -8.77675 | -16.9896 |
| TX | 2.20129 | -12.112 |
| UT | 23.2598 | 6.84776 |
| VA | 1.13169 | 0.923224 |
| VT | -1.2025 | -19.6248 |
| WA | 5.94433 | 10.6014 |
| WI | -23.3021 | -5.19293 |
| WV | 7.56142 | -4.2664 |
| WY | -4.37209 | -5.02326 |

*Note: For both 2022 and 2023, the DQ Atlas*^15^ *reported that the 1915(c) waiver HCBS participant counts were unreliable (high concern) in NC, and unusable in MO and TN.*

**MS: The % change in Mississippi was truncated for reporting purposes in this graph because the actual % change was greater than 100% due to notable changes in data quality in 2022 (6,990%) and 2023 (6,669%).*

Appendix Figure 4. Year variations in HCBS user counts (per 100,000 residents) using the Wang et al. method (Algorithm 2) for 2022 and 2023, relative to 2021

*

Annual changes in total HCBS user counts vs. 2021 using Algorithm 2 (Wang et al. method)

% Change relative to 2021

|  | 2022 | 2023 |
| --- | --- | --- |
| AK | 1.27864 | -0.586042 |
| AL | -71.6824 | -77.0989 |
| AR | 17.0544 | -0.787262 |
| AZ | 1.70915 | -7.9048 |
| CA | 6.20354 | 13.5467 |
| CO | 6.532 | 4.908 |
| CT | -2.31224 | -4.29988 |
| DC | 8.05775 | 5.16023 |
| DE | 7.51242 | 0.517384 |
| FL | -15.5438 | -7.19491 |
| GA | 3.1395 | -1.4924 |
| HI | 9.22093 | 8.4682 |
| IA | -0.418917 | -10.1753 |
| ID | 7.70393 | 4.00302 |
| IL | 14.3517 | 14.9404 |
| IN | 31.0279 | 31.6667 |
| KS | -12.6409 | 3.62932 |
| KY | 0.0925041 | -3.86667 |
| LA | 10.5954 | 10.1596 |
| MA | 0.741241 | -9.43924 |
| MD | 2.83703 | 2.8037 |
| ME | 8.30103 | 11.3372 |
| MI | 6.89324 | 0.00278122 |
| MN | 7.10659 | 13.5869 |
| MO | 0.260681 | -1.16644 |
| MS | 6343.11 | 6297.6 |
| MT | 4.39675 | -14.4885 |
| NC | -8.1907 | -13.7902 |
| ND | -5.5768 | -11.4404 |
| NE | 27.5932 | 23.4146 |
| NH | -21.4341 | -39.0781 |
| NJ | 13.3872 | 49.6223 |
| NM | 1.01004 | -2.94004 |
| NV | -0.555987 | -4.65477 |
| NY | 5.65889 | 46.8159 |
| OH | 4.52586 | -11.1215 |
| OK | 7.69485 | 9.51514 |
| OR | 41.1023 | 49.1768 |
| PA | -41.1095 | -62.4286 |
| RI | -35.9009 | -37.5214 |
| SC | 7.69306 | 4.51838 |
| SD | 15.2633 | -4.19322 |
| TN | 1.51205 | -5.40748 |
| TX | 1.80792 | -15.8364 |
| UT | 12.7403 | 33.4432 |
| VA | 4.96053 | 8.33252 |
| VT | 0.0480307 | -10.951 |
| WA | 2.78374 | 0.0174607 |
| WI | -16.7925 | -5.40186 |
| WV | 7.72057 | -3.78802 |
| WY | -3.53581 | -4.71442 |

*Note: For both 2022 and 2023, the DQ Atlas*^15^ *reported that the 1915(c) waiver HCBS participant counts were unreliable (high concern) in NC, and unusable in MO and TN.*

**MS: The % change in Mississippi was truncated for reporting purposes in this graph because the actual % change was greater than 100% due to notable changes in data quality in 2022 (6,343%) and 2023 (6,298%).*
